# Primary Total Elbow Arthroplasty With the Coonrad–Morrey Prosthesis in a Chinese Cohort: A Follow‐Up of 5–10 Years

**DOI:** 10.1111/os.70276

**Published:** 2026-03-12

**Authors:** Jianyu Zhang, Kehan Hua, Dan Xiao, Chen Chen, Maoqi Gong, Yejun Zha, Xieyuan Jiang

**Affiliations:** ^1^ Peking University Fourth School of Clinical Medicine Beijing Jishuitan Hospital Beijing China; ^2^ Department of Orthopedic Trauma, Beijing Jishuitan Hospital Capital Medical University Beijing China

**Keywords:** 5‐year survival, Chinese population, complication, Coonrad–Morrey prosthesis, rheumatoid arthritis, total elbow arthroplasty

## Abstract

**Objective:**

Total elbow arthroplasty (TEA) is a commonly performed surgical technique for the management of elbow disorders. The Coonrad–Morrey (CM) prosthesis is the most commonly used prosthesis in TEA. The study from Chinese cohorts remains limited, particularly regarding differences between patients with and without RA. Therefore, the purpose of this study was to evaluate the medium‐ to long‐term clinical outcomes using the CM prosthesis in a Chinese cohort, and to compare clinical outcomes between patients with and without RA.

**Methods:**

A retrospective cohort study was conducted involving 74 patients (75 elbows) who underwent TEA using CM prostheses between March 2015 and February 2019. All patients were followed up for a minimum of 5 years (mean follow‐up: 83.4 months) and were assessed for elbow range of motion (ROM), Mayo Elbow Performance Score (MEPS), Quick‐Disabilities of the Arm, Shoulder and Hand (Quick‐DASH) score, pain, complications, and revision surgeries. Kaplan–Meier survivorship analysis was conducted. The differences between patients with and without rheumatoid arthritis (RA) were compared in the subgroup analysis.

**Results:**

At the final follow‐up, the average flexion‐extension ROM was 105.3° ± 33.6°. The mean MEPS was 85.5 ± 14.3, with a good‐to‐excellent rate of 81.3%. The mean Quick‐DASH score was 30.8 ± 18.1. A total of 26 complications (26/75, 34.7%) were observed in 22 elbows (22/75, 29.3%). Nine elbows (9/75, 12.0%) underwent reoperation. The revision‐free rates were 98.7% at 1 year, 94.7% at 2 years, and 90.7% at 5 years. There were no significant differences in elbow function or revision‐free rate between patients with and without RA.

**Conclusion:**

TEA using CM prosthesis in Chinese patients can achieve favorable functional outcomes regardless of RA status, with a high 5‐year prosthesis survival rate. However, a larger sample size and a longer follow‐up period are still required.

## Graphical abstract

Total elbow arthroplasty is a commonly performed surgical technique for the management of elbow disorders. The Coonrad–Morrey prosthesis is the most commonly used prosthesis in total elbow arthroplasty. Total elbow arthroplasty using the Coonrad–Morrey prosthesis in Chinese patients can achieve favorable functional outcomes, with a high 5‐year prosthesis survival rate.

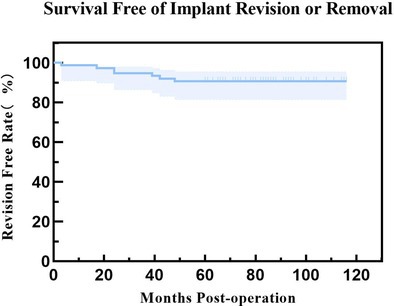

## 1 Introduction

Total elbow arthroplasty (TEA) is a commonly performed surgical technique for the management of elbow disorders [1, 2]. The primary indications for TEA include rheumatoid arthritis (RA), osteoarthritis (OA), and posttraumatic arthritis [3, 4]. RA represents a distinct clinical entity in TEA due to its systemic inflammatory nature, compromised bone quality, and soft‐tissue involvement, which may influence postoperative outcomes and prosthesis survival. With the advancement of surgical techniques, the indications have expanded to include fresh distal humeral fractures and sequelae in the elderly, as well as severe distal humeral injuries in younger patients. The Coonrad–Morrey (CM) prosthesis, a semi‐constrained hinged implant that allows a certain degree of varus‐valgus laxity, is the most widely used prosthesis [5].

Some studies have reported the medium‐ to long‐term outcomes of TEA using the CM prosthesis, indicating that patients often achieve favorable elbow function results [3, 6, 7]. The revision survival rate for CM prosthesis TEA in previous studies ranges from approximately 79% to 100% [8, 9, 10]. However, previous literature has several limitations. Many studies are based on relatively small or heterogeneous cohorts. In particular, comparative data between inflammatory conditions such as RA and noninflammatory etiologies are limited. Moreover, there is a notable lack of medium‐ to long‐term follow‐up evaluations of the CM prosthesis in the Chinese population.

Therefore, the objective of our study was to report the medium‐ to long‐term functional outcomes, complications, and prosthesis survival rates of the CM prosthesis in a Chinese cohort, and compare the differences between patients with and without RA, with a minimum follow‐up period of 5 years.

## 2 Methods and Patients

### 2.1 Study Design and Population

**FIGURE 1:**
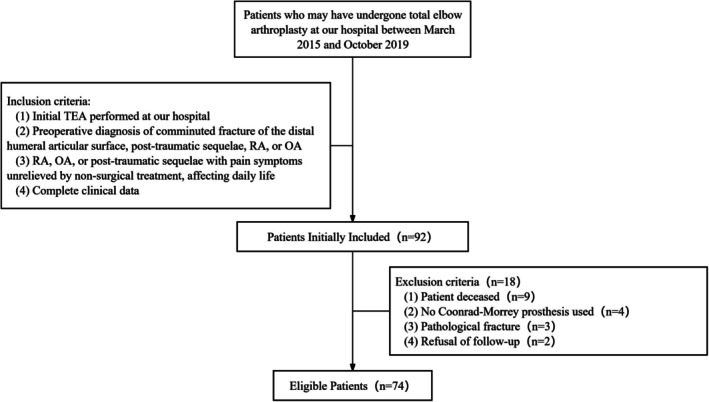
Diagram illustrating patient selection criteria.

This retrospective study aimed to report the long‐term functional outcomes, complications, and prosthesis survival rate of the CM prosthesis in a Chinese cohort. Medical records were reviewed at our hospital from March 2015 to February 2019. Samples were identified based on the following inclusion and exclusion criteria (Figure 1). Inclusion criteria: (1) Initial TEA performed at our hospital; (2) Preoperative diagnosis of comminuted fracture of the distal humeral articular surface, posttraumatic sequelae (PTS), RA, or OA; (3) RA, OA, or PTS with pain symptoms unrelieved by nonsurgical treatment, affecting daily life; and (4) complete clinical data. Exclusion criteria: (1) patient deceased; (2) pathological fracture; (3) no CM prosthesis used; and (4) refusal of follow‐up. In the subgroup analysis, patients were grouped based on whether patients had RA; specifically, those with acute distal humeral fractures (ADHFs), OA, and PTS were assigned to the non‐RA group. This study received ethical approval from the Ethics Committee of Beijing Jishuitan Hospital, Capital Medical University, in compliance with the Declaration of Helsinki and local regulations (Approval No. K2024‐031‐00). Written informed consent was obtained from all the participants prior to the enrollment of this study.

### 2.2 Surgical Procedure

**FIGURE 2:**
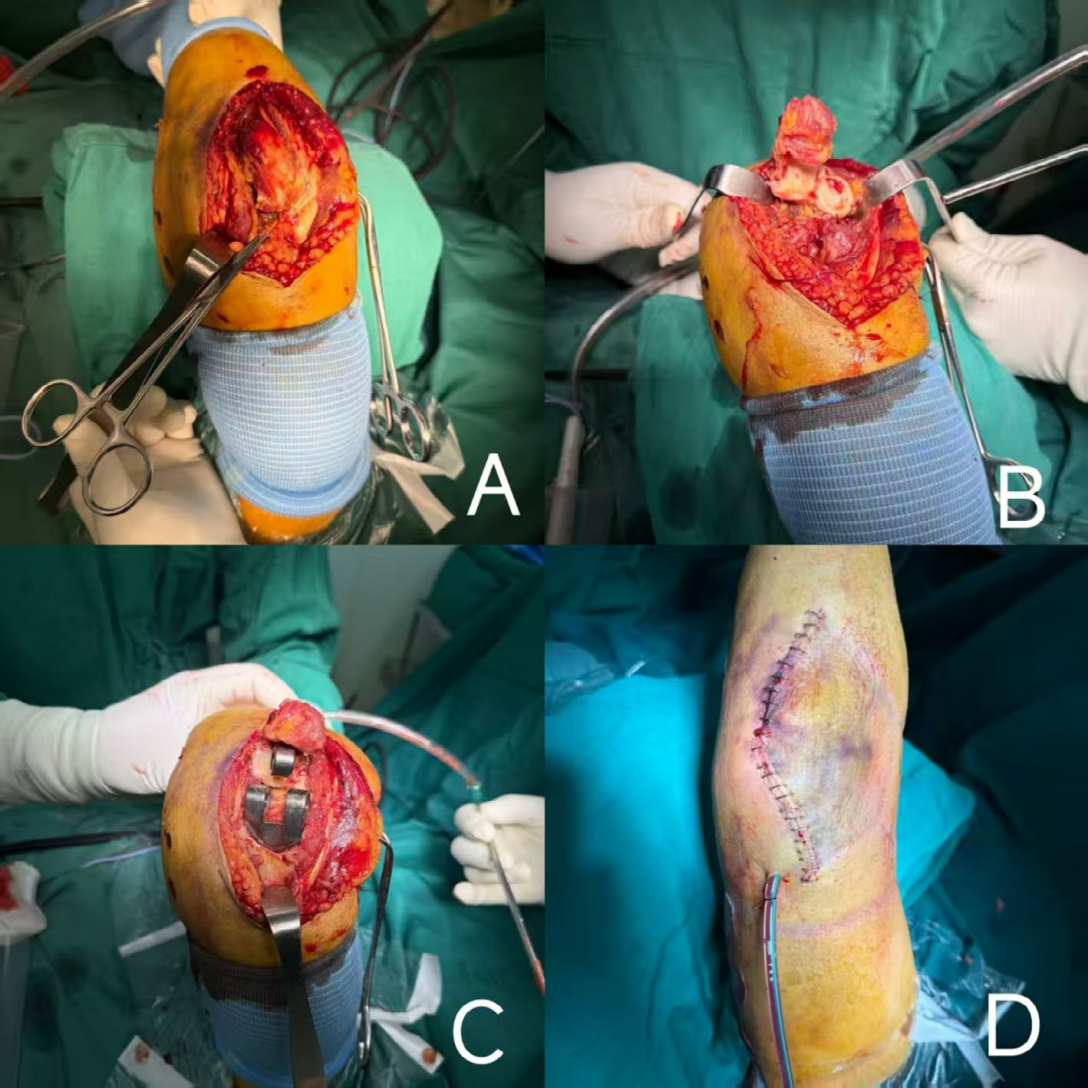
Intraoperative photographs of the modified triceps fascial tongue approach. (A) Make a rhomboid incision through the medial and lateral soft tissues of the olecranon and the triceps brachii muscle. (B) Dislocate the ulna and radius. (C) Implant the prosthesis. (D) Place the drainage tube and close the incision.

Brachial plexus anesthesia was administered, with some patients also receiving general anesthesia. Patients were positioned supine with the upper limb placed on the chest for the operation. A posterior midline incision was typically utilized. Three common approaches were used: (1) Bryan–Morrey approach [11, 12]: The triceps tendon was completely detached from the olecranon; (2) triceps‐preserving approach [13, 14]: The operation was performed from both sides of the triceps; (3) modified triceps facial tongue approach [15]: A posterior midline elbow incision was adopted with medial and lateral fasciocutaneous flaps dissected to the anterior margins of the epicondyles. Only 2 cm‐long flaps were dissected on both the medial and lateral sides of the olecranon tip. A diamond‐shaped incision was made through the medial and lateral soft tissues of the olecranon and the triceps brachii muscle (Figure 2A). The tongue‐shaped flap of the triceps brachii was reflected distally and secured with sutures.

Dislocate the ulna and radius, and resect the tips of the olecranon and coronoid process to prevent postoperative impingement on the prosthesis (Figure 2B). The radial head was generally excised for patients with RA. The middle portion of the humeral trochlea was resected, and the distal humerus was exposed anteriorly using an osteotomy guide to accommodate the humeral wing. On the ulnar side, the medullary canal was opened. The trial components were connected to assess the depth of prosthesis stem insertion, range of motion (ROM), stability of the elbow, and confirm the position of the trial components. The medullary cavity was irrigated and dried, and antibiotic‐loaded bone cement was injected using a cement gun. Excess cement was removed, and the prosthesis was implanted (Figure 2C). The implant size was typically small or X‐small. Heavy absorbable sutures were used for reinforced suture fixation of the tendinous portion of the triceps brachii muscle. The range of flexion and extension movements, as well as the continuity and tension of the triceps brachii muscle, were inspected. If necessary, additional reinforced sutures could be performed. A drainage tube was placed around the prosthesis and in the subcutaneous layer, followed by layered closure of the incision (Figure 2D).

### 2.3 Postoperative Rehabilitation

All patients began functional exercises on the second postoperative day, performing flexion and extension with assistance from the unaffected hand, while avoiding active movements and forceful passive elbow flexion. A drainage tube was placed postoperatively and removed when drainage was less than 30 mL/day after sufficient activity. The maximum weight‐bearing capacity of the affected limb should not exceed 5 kg, and repetitive weight‐bearing should not exceed 1 kg.

### 2.4 Follow Up

Patients were routinely followed on an annual basis after surgery, with a minimum follow‐up duration of 5 years and a maximum of 10 years. The MEPS, quick‐DASH scores and Visual Analog Scale (VAS) were documented and the elbow ROM, including extension, flexion, supination, and pronation were measured. The measurement was conducted by a single observer. Complications including ulnar nerve symptoms, periprosthetic joint infections (PJIs), aseptic loosening, periprosthetic fractures (PFs), and reoperations were also recorded. Kaplan–Meier curves were used to calculate the reoperation and revision survival rates.

### 2.5 Statistical Analysis

Data were processed and analyzed using SPSS 25.0 statistical software (IBM Corp., USA). The normality of continuous data was evaluated using the Shapiro–Wilk test, and the Levene test was applied to assess homogeneity of variance. Normally distributed data are presented as the mean ± standard deviation (*x¯* ± *s*), while non‐normally distributed data are expressed as *M* (*P*_25_, *P*_75_). If variance was homogeneous, an independent sample *t*‐test was conducted. For data that did not follow a normal distribution, we employed the Mann–Whitney *U* test. Categorical variables were analyzed with Pearson's chi‐square test or Fisher's exact test for comparisons between two groups. A *p* < 0.05 was considered statistically significant in our study.

## 3 Results

### 3.1 Demographic Data

This study involved a continuous retrospective analysis of 74 patients (75 elbows) with a mean follow‐up period of 83.4 ± 15.2 months (ranging from 5 to 10 years). The mean age of the patients was 64.3 ± 11.3 years, with a range from 35 to 89 years. The cohort consisted of 17 males and 57 females (Table 1). In the subgroup analysis, there were 59 elbows in the non‐RA group and 16 elbows in the RA group. Baseline data were basically comparable, except that the age in the non‐RA group was significantly higher than that in the RA group (66.2 ± 10.6 vs. 57.2 ± 11.2, *p* < 0.01).

**TABLE 1:** Patients demographic data and intraoperative management.

	Total (*N* = 75)	Non‐RA (*N* = 59)	RA (*N* = 16)	*p*
Demographic data				
Age (year)^a^	64.3 ± 11.3	66.2 ± 10.6	57.2 ± 11.2	**0.004**
Gender (no. [%])				0.15
Male	18 (24.0%)	12 (20.3%)	6 (37.5%)	
Female	57 (76.0%)	47 (79.7%)	10 (62.5%)	
Weight (kg)^a^	64.5 ± 12.0	64.4 ± 9.3	65.0 ± 19.6	0.91
Height (m)^a^	1.6 ± 0.1	1.6 ± 0.1	1.7 ± 0.1	0.33
BMI(kg/m^2^)^a^	24.0 ± 3.3	24.3 ± 3.1	23.0 ± 4.1	0.18
Intraoperative management				
Radial head resection (no. [%])				**0.0001**
Yes	16 (21.3%)	4 (6.8%)	12 (75.0%)	
No	59 (78.7%)	55 (93.2%)	4 (75.0%)	
Condyle preservation (no. [%])				0.64
Yes	20 (26.7%)	15 (25.4%)	5 (31.2%)	
No	55 (73.3%)	44 (74.6%)	11 (68.8%)	
Ulnar nerve management (no. [%])				0.41
Situ decompression	40 (53.3%)	30 (50.8%)	10 (62.5%)	
Anterior transposition	35 (46.7%)	29 (49.2%)	6 (37.5%)	
Surgical duration (min)^a^	124.3 ± 35.1	126.9 ± 36.2	114.7 ± 29.6	0.22
Total hospital stay (day)^a^	10.2 ± 3.5	10.3 ± 3.7	9.8 ± 2.6	0.57
Postoperative hospital stay (day)^a^	4.7 ± 1.4	4.8 ± 1.4	4.4 ± 1.4	0.44

*Note:* Both bold values represent statistically significant differences.

Abbreviation: RA, rheumatoid arthritis.

^a^ The values are given as the mean and standard deviation.

### 3.2 Intraoperative Management

In terms of surgical approaches, 62 elbows were treated with a triceps‐preserving technique, 8 with a modified triceps fascial tongue approach, 3 with an olecranon osteotomy due to concomitant olecranon fractures, and 2 with the Bryan–Morrey approach. During surgery, 16 elbows (16/75, 21.3%) underwent radial head resection, and 20 elbows (20/75, 26.7%) underwent condyle preservation. Regarding ulnar nerve management, 40 elbows underwent in situ decompression, while 35 elbows underwent anterior transposition. The mean surgical duration was 124.3 ± 35.1 min. The mean total hospital stay was 10.2 ± 3.5 days, with a mean postoperative stay of 4.7 ± 1.4 days. No differences were observed in intraoperative management between the two groups, except that a higher rate of radial head resection was performed in the RA group (75.0% vs. 6.8%, *p* < 0.01).

### 3.3 Elbow Function

At the final follow‐up, the mean flexion‐extension ROM of the elbow was 105.3° ± 33.6°, and the mean pronation‐supination ROM was 138.1° ± 39.2°. The mean extension was 21.4° ± 23.8°, flexion was 126.7° ± 14.7°, pronation was 67.7° ± 19.3°, and supination was 70.3° ± 21.4°. The mean MEPS was 85.5 ± 14.3, with 40 patients rated as excellent, 21 as good, 12 as fair, and 2 as poor, resulting in an excellent‐good rate of 81.3% (61/75). The mean Quick‐DASH score was 30.8 ± 18.1. The average VAS for all patients was 1.2 ± 1.8 (Table 2). No differences in elbow function were observed between the non‐RA group and the RA group.

**TABLE 2:** Patient' clinical outcomes.

	Total (*N* = 75)	Non‐RA (*N* = 59)	RA (*N* = 16)	*p*
Flexion‐extension ROM (°)^a^	105.3 ± 33.6	107.0 ± 31.5	99.4 ± 41.1	0.43
Flexion (°)^a^	126.7 ± 14.7	126.0 ± 15.0	129.4 ± 13.3	0.42
Extension (°)^a^	21.4 ± 23.8	19.1 ± 20.3	30.0 ± 33.3	0.10
Pronation–supination ROM (°)^a^	138.1 ± 39.2	137.8 ± 37.0	139.1 ± 47.7	0.91
Pronation (°)^a^	67.7 ± 19.3	68.1 ± 17.6	66.3 ± 25.3	0.73
Supination (°)^a^	70.3 ± 21.4	69.7 ± 20.6	72.8 ± 24.8	0.61
MEPS^a^	85.5 ± 14.3	85.0 ± 14.0	87.5 ± 15.9	0.54
Quick‐DASH^a^	30.8 ± 18.1	31.9 ± 17.8	26.4 ± 19.1	0.28
VAS^a^	1.2 ± 1.8	1.4 ± 1.9	0.6 ± 1.2	0.13
Total complications (no. [%])	26 (34.7%)	21 (35.6%)	5 (31.3%)	0.75
Reoperation‐free rate				0.43
1 year	97.3%	96.6%	100.0%	
2 years	93.3%	91.5%	100.0%	
5 years	88.0%	86.4%	93.8%	
The revision‐free rate				0.63
1 year	98.7%	98.3%	100.0%	
2 years	94.7%	93.2%	100.0%	
5 years	90.7%	89.8%	93.8%	

Abbreviations: MEPS, Mayo Elbow Performance Score; Quick‐DASH, Quick‐Disabilities of the Arm, Shoulder and Hand; RA, rheumatoid arthritis; VAS, Visual Analog Scale.

^a^ The values are given as the mean and standard deviation.

### 3.4 Complications

A total of 26 complications (26/75, 34.7%) occurred in 22 elbows (22/75, 29.3%). Sixteen elbows (16/75, 21.3%) developed ulnar nerve symptoms postoperatively, with eight classified as modified McGowan Grade 1, seven as Grade 2, and one as Grade 3. The incidence of unresolved ulnar nerve symptoms at the final follow‐up was 10.7% (8/75). Four elbows developed PJI, three experienced aseptic loosening, two presented with PF, and one encountered prosthetic mechanical complication. There were no significant differences in total complications between the non‐RA group and the RA group.

### 3.5 Reoperations

**FIGURE 3:**
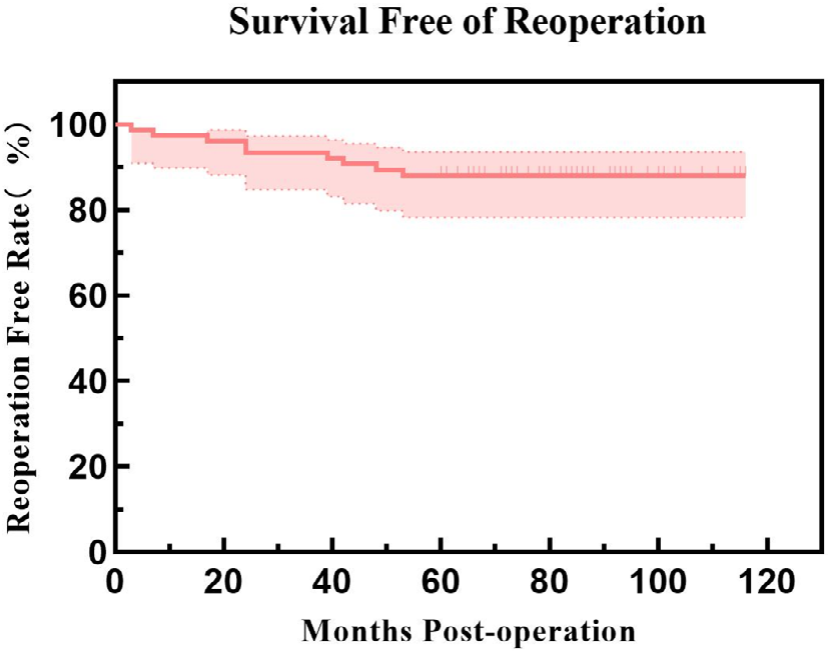
Kaplan–Meier curve for reoperation‐free survival, showing a 5‐year reoperation‐free survival rate of 88.0%.

**FIGURE 4:**
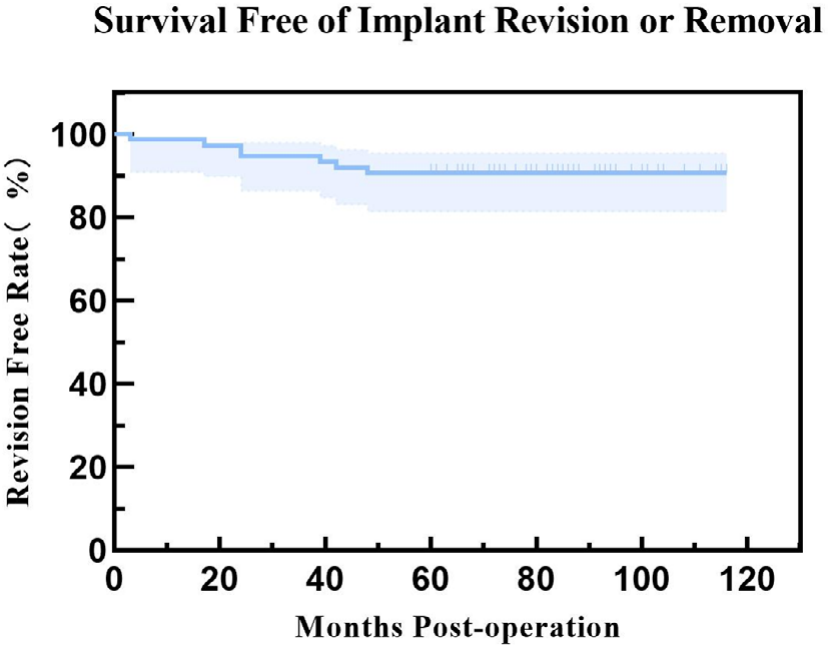
Kaplan–Meier curve for implant revision‐free survival, showing a 5‐year revision‐free survival rate of 90.7%.

**FIGURE 5:**
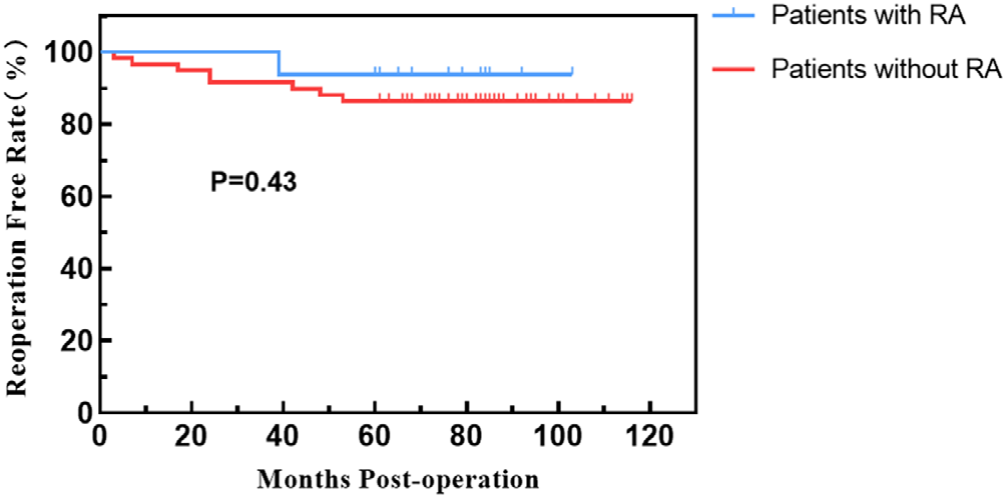
Kaplan–Meier curve for reoperation‐free survival of subgroups, showing no significant difference between the RA and non‐RA groups (*p* = 0.43).

**FIGURE 6:**
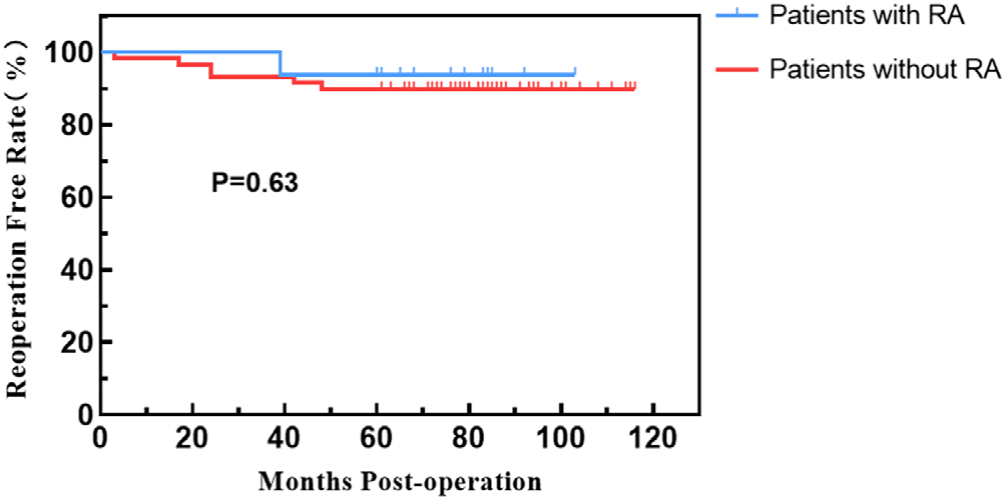
Kaplan–Meier curve for implant revision‐free survival of subgroups, showing no significant difference between the RA and non‐RA groups (*p* = 0.63).

**FIGURE 7:**
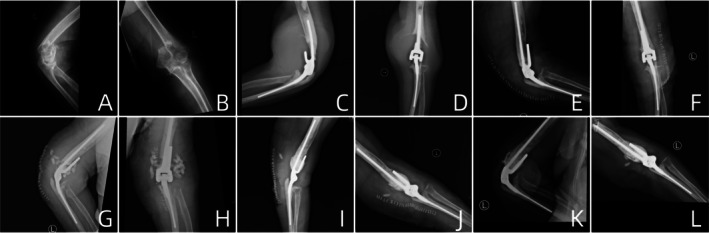
A typical case of a 77‐year‐old female: (A–D) TEA was performed for ADHF; (E, F) secondary surgery was performed for elbow swelling, with humeral component revision and lesion debridement due to identified prosthetic loosening; (G, H) debridement with implant retention and antibiotic‐loaded bone cement beads implantation for PJI; (I, J) bead removal and local antibiotic‐loaded bone cement augmentation; (K, L) follow‐up: no prosthetic loosening identified, infection well controlled.

A total of 9 elbows (9/75, 12.0%) underwent reoperation, with further details available in Table 3. The reoperation‐free rates were 97.3% at 1 year, 93.3% at 2 years, and 88.0% at 5 years (Figure 3). The revision‐free rates were 98.7% at 1 year, 94.7% at 2 years, and 90.7% at 5 years (Figure 4). Four elbows underwent reoperation due to PJI, with two elbows achieving infection control after debridement while retaining the prosthesis, and two elbows requiring multiple debridement and prosthesis revision (Figures 5 and 6). Among the four infected elbows, one elbow was ultimately arthrodesed in a functional position, whereas the remaining three elbows recovered functional ROM without limitation in daily activities (Figure 7, typical case). Two elbows underwent revision surgery due to PF. Postoperatively, one patient experienced mild elbow stiffness, while the other regained normal elbow motion. Two elbows underwent revision surgery for aseptic loosening of the prosthesis. Both achieved functional ROM at final follow‐up; however, one patient demonstrated mild elbow instability. One elbow underwent elbow arthrolysis for elbow stiffness, resulting in functional elbow motion without impairment of daily living.

**TABLE 3:** Details of reoperation patients.

Case no.	Age (year)	Gender	Surgical indication	Interval before reoperation (months)	Specific surgical methods
1	83	Female	ADHF	53	One debridement for PJI, retaining the prosthesis
2	59	Male	RA	39	Polyethylene liner and pin exchange, followed by four debridement, prosthesis removal, and spacer placement due to PJI
3	51	Male	PTS	3	Prosthesis revision for PF with aseptic loosening
4	55	Male	OA	48	Four debridement, prosthesis removal, and spacer placement due to PJI
5	77	Female	ADHF	42	Prosthesis revision for loosening, followed by two debridement retaining the prosthesis due to PJI
6	62	Female	ADHF	7	Open elbow release for intolerable elbow stiffness
7	35	Male	PTS	24	Prosthesis revision for PF
8	68	Male	OA	24	Prosthesis revision for aseptic loosening
9	55	Female	OA	17	Prosthesis revision for aseptic loosening

Abbreviations: ADHF, acute distal humeral fracture; OA, osteoarthritis; PF, periprosthetic fracture; PJI, periprosthetic joint infection; PTS, posttraumatic sequelae; RA, rheumatoid arthritis.

## 4 Discussion

### 4.1 Functional Outcomes and Prosthesis Survivorship

In this study, TEA using the CM prosthesis achieved satisfactory functional outcomes in a Chinese cohort, with a 5‐year prosthesis survival rate of 90.7%. Comparable outcomes were observed in patients with and without RA. These findings directly address the primary objective of the study and provide mid‐ to long‐term evidence supporting the durability of the CM prosthesis in Chinese patients.

TEA is widely recognized for significantly improving patients' flexion‐extension ROM and enhancing joint stability [16, 17, 18, 19]. The CM prosthesis, as the most extensively utilized implant, has demonstrated favorable clinical outcomes validated by multiple studies [10, 20, 21]. However, current research on the application of CM prostheses in Chinese patients is often limited by short follow‐up duration and small sample size [12, 22]. Our 5‐year survivorship of 90.7% is consistent with previously reported results. Tarallo et al. conducted a study with an average 9.9‐year follow‐up on 122 elbow joints, reporting a 5‐year prosthesis survival rate of 90.2%, with aseptic loosening being the most common reason for revision (11/122, 9.0%) [23]. Another retrospective study reported on 63 patients aged ≤ 45 years who underwent TEA for PTS, showing a 5‐year prosthesis survival rate of 79.4%, with PJI being the most common reason for revision (6/63, 9.5%) [8]. A systematic review summarized the 5‐year prosthesis survival rates from 12 studies, which ranged between 85% and 100% [10]. In our study, the 5‐year prosthesis survival rate was 90.7%, which is consistent with the results of previous studies.

### 4.2 Comparison Between RA and Non‐RA Patients

One of the primary aims of this study was to compare outcomes between patients with and without RA. In the subgroup analysis grouped by RA, no significant differences were observed in terms of elbow function, complications, and reoperations, indicating that RA itself may not be a determinant of postoperative outcomes in this cohort. Barco et al. [1] reported similar findings in a cohort of 44 patients undergoing TEA for distal humeral fractures, demonstrating minimal functional differences between RA and non‐RA patients. Consistent with our results, patients in the RA group were significantly younger (*p* < 0.01), which may reflect a lower threshold for selecting TEA over internal fixation in RA patients due to compromised bone quality and joint pathology. Their study also demonstrated minimal differences in postoperative elbow function between RA and non‐RA patients, further supporting our results. However, the revision‐free rate in the non‐RA group was higher than that in the RA group in their study, whereas no such difference was observed in ours. This observed discrepancy might be explained by our enrollment of patients with complex indications in the non‐RA group, together with a follow‐up duration that has not yet been extended sufficiently.

### 4.3 Complications and Causes of Reoperation

Another objective of this study was to evaluate complications and reoperations after CM TEA. PJI remains one of the most challenging complications after TEA, with reported incidences ranging from 1.2% to 11.7% [24, 25]. The elbow is particularly vulnerable to infection because of limited soft tissue coverage and the high proportion of patients with inflammatory disease undergoing TEA. Treatment modalities primarily involve the use of long‐acting antibiotics, early and thorough debridement, and prosthesis revision [26, 27, 28]. In our study, the incidence rate of PJI was 5.3% (4/75), making it the most common cause of reoperation. Among these cases, three required multiple debridement surgeries, and two underwent prosthesis revision due to PJI, one of whom had RA.

Ulnar nerve symptom is another common complication following TEA [29]. Although most researchers believe that the ulnar nerve should be clearly exposed and protected during TEA [30, 31], the optimal management strategy remains controversial. Reported incidences of postoperative ulnar nerve symptoms vary widely between in situ decompression and routine transposition. Little et al. conducted a systematic review of 2416 TEA cases and found that the incidence of permanent ulnar nerve lesions was 5% [32]. Dachs et al. analyzed 78 TEA patients who underwent in situ decompression, among which 4 (5.1%) patients developed ulnar nerve symptoms [33]. Hildebrand et al. reported on 39 TEA patients who underwent ulnar nerve transposition, with 10 (25.6%) experiencing ulnar nerve symptoms, of which 4 (10.3%) were permanent [34]. According to a systematic review by Voloshin et al., the incidence of ulnar nerve symptoms after routine transposition was 2.0%, while the complication rate for in situ decompression was 3.2%, with no statistically significant difference (*p* > 0.5) [35]. In our study, the incidence of unresolved ulnar nerve symptoms was 10.7%. The authors believe that the decision to transpose the ulnar nerve should depend on intraoperative conditions. The general principle emphasizes the need for meticulous intraoperative technique to protect the ulnar nerve, avoid postoperative tension, prevent direct contact between the ulnar nerve and prosthesis, and avoid friction during elbow flexion and extension.

### 4.4 Surgical Technique Considerations

Surgical technique represents an important factor influencing outcomes after TEA. Different approaches offer varying balances between exposure and preservation of the extensor mechanism. The commonly used Bryan–Morrey approach provides good intraoperative visibility but necessitates detaching the triceps from the olecranon, which may lead to complications such as ulnar nerve symptoms and triceps weakness. The triceps‐preserving approach avoids postoperative triceps weakness and has gained widespread acceptance. However, it still requires complete mobilization of the ulnar nerve and extensive dissection in patients with well‐developed triceps muscles. The modified triceps tongue facial approach is a novel technique that involves a “V‐shaped” incision of the triceps, followed by tendon‐to‐tendon repair and reinforcement with surrounding fascia. This approach only necessitates opening the epineurium of the ulnar nerve for decompression. Na et al. retrospectively analyzed 21 patients who underwent the modified triceps fascia tongue approach. Triceps strength was normal (MRC Grade V) in 10 elbows and good (MRC Grade IV) in 11. The elbow ROM improved from 78° preoperatively to 100° postoperatively (*p* = 0.004) [15].

The management of the humeral condyles remains another technical consideration. It remains unclear whether resection of medial and lateral humeral condyles affects muscle strength, elbow function, and stability in TEA patients. McKee et al. investigated the impact of condyle resection in 32 patients with an average age of 67 years, who underwent TEA using CM prosthesis. Humeral condyles were preserved in 16 cases and resected in 16 cases. After an average follow‐up of 64 months, no significant differences were found in upper limb strength and elbow function between the two groups [36]. Celli et al. included 13 patients with ADHF treated with CM prosthesis, of whom 7 underwent condyle resection and 6 had condyle preservation. After a 1‐year follow‐up, no significant differences in elbow function were observed between the two groups [37]. The author contends that condyle resection subjects the hinge to greater mechanical stress, thereby increasing the risk of bushing wear. Condyles retention using plates or Kirschner wires appears to provide longer prosthesis survival and is recommended for younger patients with higher functional demands. However, large‐scale prospective randomized controlled studies are still needed to confirm these findings. In our study, humeral condyles were preserved in 20 (20/75, 26.7%) patients. The authors recommend preserving the humeral condyles whenever possible. If preservation is not feasible due to comminuted fractures, condyle resection should be performed.

### 4.5 Strengths and Limitations

This study has several strengths. This study provides medium‐ to long‐term follow‐up data on TEA using the CM prosthesis in a Chinese cohort. Comprehensive evaluation of clinical outcomes was performed at a single center using standardized surgical and rehabilitation protocols. In addition, direct comparison between patients with and without RA allowed focused assessment of the influence of RA status on outcomes. There are several limitations in our study. First, the exclusion of deceased patients and the inability to obtain clinical data may have introduced bias in the results. Second, radiological results for some patients were unavailable in the system, so radiological findings were not reported. Furthermore, the minimum follow‐up duration in this study was 5 years, and the follow‐up period should be extended in future research.

## 5 Conclusion

This study demonstrates that TEA using the CM prosthesis provides reliable medium‐ to long‐term functional outcomes and satisfactory prosthesis survivorship in a Chinese population. No significant differences were identified between patients with and without RA in clinical outcomes, suggesting that RA status may not be a major determinant of mid‐term outcomes in this cohort. PJI remained the leading cause of reoperation, highlighting the importance of meticulous surgical technique and postoperative management.

## Author Contributions

Jianyu Zhang and Kehan Hua contributed equally to this work. Jianyu Zhang contributed to conceptualization, methodology, data curation, formal analysis, visualization, and writing. Kehan Hua contributed to data curation, validation, formal analysis, visualization, and writing. Dan Xiao contributed to conceptualization, methodology, data curation, validation, and formal analysis. Chen Chen contributed to conceptualization, methodology, formal analysis, visualization, supervision, and resources. Maoqi Gong contributed to supervision, project administration, visualization, and resources. Yejun Zha contributed to formal analysis, supervision, funding acquisition, visualization, project administration, resources, and writing. Xieyuan Jiang contributed to supervision, funding acquisition, visualization, project administration, and resources.

## Funding

This research was supported by Beijing Natural Science Foundation (L244014), Beijing Jishuitan Hospital Research Fund (QN202506), Young Elite Scientists Sponsorship Program by Beijing Association for Science and Technology (No. BYESS2023115).

## Conflicts of Interest

The authors declare no conflicts of interest.

## Data Availability

The data that support the findings of this study are available on request from the corresponding author. The data are not publicly available due to privacy or ethical restrictions.

## References

[1] R. Barco , P. N. Streubel , B. F. Morrey , and J. Sanchez‐Sotelo , “Total Elbow Arthroplasty for Distal Humeral Fractures: A Ten‐Year‐Minimum Follow‐Up Study,” Journal of Bone and Joint Surgery. American Volume 99, no. 18 (2017): 1524–1531. 28926381 10.2106/JBJS.16.01222

[2] D. Zhang and N. Chen , “Total Elbow Arthroplasty,” Journal of Hand Surgery 44, no. 6 (2019): 487–495. 30635202 10.1016/j.jhsa.2018.11.005

[3] T. A. Chou , H. H. Ma , J. H. Wang , et al., “Total Elbow Arthroplasty in Patients With Rheumatoid Arthritis,” Bone & Joint Journal 102‐b, no. 8 (2020): 967–980. 10.1302/0301-620X.102B8.BJJ-2019-1465.R1 PMC7954192 32731835

[4] B. S. Schoch , J. D. Werthel , J. Sánchez‐Sotelo , et al., “Total Elbow Arthroplasty for Primary Osteoarthritis,” Journal of Shoulder and Elbow Surgery 26, no. 8 (2017): 1355–1359. 28734537 10.1016/j.jse.2017.04.003

[5] E. Kholinne , L. A. Altamimi , A. Aldayel , et al., “Primary Linked Total Elbow Arthroplasty for Acute Distal Humerus Fracture Management: A Systematic Review of Clinical Outcome,” Clinics in Orthopedic Surgery 12, no. 4 (2020): 503–513. 33274028 10.4055/cios20012 PMC7683186

[6] P. Y. Barthel , P. Mansat , F. Sirveaux , F. Dap , D. Molé , and G. Dautel , “Is Total Elbow Arthroplasty Indicated in the Treatment of Traumatic Sequelae? 19 Cases of Coonrad–Morrey Reviewed at a Mean Follow‐Up of 5.2 Years,” Orthopaedics & Traumatology, Surgery & Research 100, no. 1 (2014): 113–118. 10.1016/j.otsr.2013.10.012 24370486

[7] A. Celli and B. F. Morrey , “Total Elbow Arthroplasty in Patients Forty Years of Age or Less,” Journal of Bone and Joint Surgery. American Volume 91, no. 6 (2009): 1414–1418. 19487519 10.2106/JBJS.G.00329

[8] A. G. Aliyev , R. M. Tikhilov , I. I. Shubnyakov , et al., “Coonrad‐Morrey Total Elbow Arthroplasty Implications in Young Patients With Post‐Traumatic Sequelae,” Journal of Shoulder and Elbow Surgery 31, no. 9 (2022): 1874–1883. 35533979 10.1016/j.jse.2022.03.021

[9] D. R. Gill and B. F. Morrey , “The Coonrad‐Morrey Total Elbow Arthroplasty in Patients Who Have Rheumatoid Arthritis. A Ten to Fifteen‐Year Follow‐Up Study,” Journal of Bone and Joint Surgery. American Volume 80, no. 9 (1998): 1327–1335. 9759818 10.2106/00004623-199809000-00012

[10] M. Morandi Guaitoli , A. Mazzotti , E. Artioli , A. Arceri , A. Ruffilli , and C. Faldini , “Indications and Outcomes of the Coonrad‐Morrey Total Elbow Arthroplasty: A Systematic Review,” Archives of Orthopaedic and Trauma Surgery 145, no. 1 (2024): 19. 39666066 10.1007/s00402-024-05636-4

[11] B. F. Morrey and J. Sanchez‐Sotelo , “Approaches for Elbow Arthroplasty: How to Handle the Triceps,” Journal of Shoulder and Elbow Surgery 20, no. 2 Suppl (2011): S90–S96. 21281925 10.1016/j.jse.2010.12.004

[12] Z. Xue , X. Huang , W. Guo , et al., “Comparison of Clinical Outcomes Between the Olecranon Osteotomy Approach and the Bryan–Morrey Approach for Total Elbow Arthroplasty,” Journal of Shoulder and Elbow Surgery 32, no. 7 (2023): 1505–1513. 36958523 10.1016/j.jse.2023.02.128

[13] T. D. Pierce and J. H. Herndon , “The Triceps Preserving Approach to Total Elbow Arthroplasty,” Clinical Orthopaedics and Related Research 354 (1998): 144–152. 10.1097/00003086-199809000-00017 9755773

[14] P. M. Prokopis and A. J. Weiland , “The Triceps‐Preserving Approach for Semiconstrained Total Elbow Arthroplasty,” Journal of Shoulder and Elbow Surgery 17, no. 3 (2008): 454–458. 18359644 10.1016/j.jse.2008.02.002

[15] K. T. Na , S. W. Song , Y. M. Lee , and J.‐H. Choi , “Modified Triceps Fascial Tongue Approach for Primary Total Elbow Arthroplasty,” Journal of Shoulder and Elbow Surgery 27, no. 5 (2018): 887–893. 29496333 10.1016/j.jse.2018.01.005

[16] R. P. Dachs , M. A. Fleming , D. A. Chivers , et al., “Total Elbow Arthroplasty: Outcomes After Triceps‐Detaching and Triceps‐Sparing Approaches,” Journal of Shoulder and Elbow Surgery 24, no. 3 (2015): 339–347. 25591460 10.1016/j.jse.2014.11.038

[17] D. Meijering , A. L. Boerboom , C. L. E. Gerritsma , et al., “Mid‐Term Results of the Latitude Primary Total Elbow Arthroplasty,” Journal of Shoulder and Elbow Surgery 31, no. 2 (2022): 382–390. 34619349 10.1016/j.jse.2021.08.028

[18] N. Mühlenfeld , I. Marzi , and J. Frank , “Total Elbow Arthroplasty in Elderly Trauma Patients: Adding a New Perspective for Functional Evaluation,” European Journal of Trauma and Emergency Surgery 48, no. 5 (2022): 3941–3947. 35246702 10.1007/s00068-022-01921-2 PMC9532322

[19] D. W. Zeltser , H. A. Prentice , R. A. Navarro , R. Mirzayan , M. T. Dillon , and A. Foroohar , “Total Elbow Arthroplasty: A Descriptive Analysis of 170 Patients From a United States Integrated Health Care System,” Journal of Hand Surgery 46, no. 7 (2021): 552–559. 10.1016/j.jhsa.2021.03.005 33896647

[20] H. Barret , P. Laumonerie , S. Delclaux , M. Arboucalot , N. Bonnevialle , and P. Mansat , “Revision Total Elbow Arthroplasty With the Semiconstrained Coonrad/Morrey Prosthesis: Follow‐Up to 21 Years,” Journal of Bone and Joint Surgery. American Volume 103, no. 7 (2021): 618–628. 33617163 10.2106/JBJS.20.00889

[21] M. Siala , P. Laumonerie , A. Hedjoudje , et al., “Outcomes of Semiconstrained Total Elbow Arthroplasty Performed for Arthritis in Patients Under 55 Years Old,” Journal of Shoulder and Elbow Surgery 29, no. 4 (2020): 859–866. 31629652 10.1016/j.jse.2019.08.006

[22] Q. Zhang , M. Xiang , J. S. Yang , et al., “Clinical and Radiographic Outcomes of Total Elbow Arthroplasty Using a Semi‐Constrained Prosthesis With a Triceps‐Preserving Approach Over a Minimum Follow‐Up Period of 4 Years,” Orthopaedic Surgery 15, no. 8 (2023): 2091–2101. 37076437 10.1111/os.13698 PMC10432419

[23] L. Tarallo , A. Celli , M. Delvecchio , et al., “Long‐Term Outcomes and Trends in Elbow Arthroplasty With Coonrad‐Morrey Prosthesis: A Retrospective Study in Large Group of Patients,” International Orthopaedics 48, no. 10 (2024): 2689–2698. 39172271 10.1007/s00264-024-06272-8 PMC11422475

[24] N. Goyal , T. J. Luchetti , R. W. Wysocki , and M. S. Cohen , “Management of Periprosthetic Joint Infection in Total Elbow Arthroplasty,” Journal of Hand Surgery 45, no. 10 (2020): 957–970. 32753227 10.1016/j.jhsa.2020.05.020

[25] M. J. Gutman , M. A. Stone , S. Namdari , and J. A. Abboud , “Treatment of Elbow Periprosthetic Joint Infection: A Systematic Review of Clinical Outcomes,” Journal of Shoulder and Elbow Surgery 29, no. 2 (2020): 411–419. 31952561 10.1016/j.jse.2019.10.002

[26] N. Martinez‐Catalan , N. T. V. Nguyen , M. E. Morrey , et al., “Two‐Stage Reimplantation for Deep Infection After Total Elbow Arthroplasty,” Shoulder & Elbow 14, no. 6 (2022): 668–676. 36479006 10.1177/17585732211043524 PMC9720873

[27] W. B. J. Rudge , K. Eseonu , M. Brown , et al., “The Management of Infected Elbow Arthroplasty by Two‐Stage Revision,” Journal of Shoulder and Elbow Surgery 27, no. 5 (2018): 879–886. 29503100 10.1016/j.jse.2017.12.033

[28] B. Zmistowski , A. Pourjafari , E. M. Padegimas , et al., “Treatment of Periprosthetic Joint Infection of the Elbow: 15‐Year Experience at a Single Institution,” Journal of Shoulder and Elbow Surgery 27, no. 9 (2018): 1636–1641. 30045830 10.1016/j.jse.2018.05.035

[29] J. M. Kwak , K. H. Koh , and I. H. Jeon , “Total Elbow Arthroplasty: Clinical Outcomes, Complications, and Revision Surgery,” Clinics in Orthopedic Surgery 11, no. 4 (2019): 369–379. 31788158 10.4055/cios.2019.11.4.369 PMC6867907

[30] A. Choo and M. L. Ramsey , “Total Elbow Arthroplasty: Current Options,” Journal of the American Academy of Orthopaedic Surgeons 21, no. 7 (2013): 427–437. 23818030 10.5435/JAAOS-21-07-427

[31] J. Sanchez‐Sotelo and B. F. Morrey , “Total Elbow Arthroplasty,” Journal of the American Academy of Orthopaedic Surgeons 19, no. 2 (2011): 121–125. 21292935 10.5435/00124635-201102000-00007

[32] C. P. Little , A. J. Graham , and A. J. Carr , “Total Elbow Arthroplasty: A Systematic Review of the Literature in the English Language Until the End of 2003,” Journal of Bone and Joint Surgery. British Volume 87, no. 4 (2005): 437–444. 15795188 10.1302/0301-620X.87B4.15692

[33] R. P. Dachs , B. C. Vrettos , D. A. Chivers , et al., “Outcomes After Ulnar Nerve In Situ Release During Total Elbow Arthroplasty,” Journal of Hand Surgery 40, no. 9 (2015): 1832–1837. 26254945 10.1016/j.jhsa.2015.06.107

[34] K. A. Hildebrand , S. D. Patterson , W. D. Regan , et al., “Functional Outcome of Semiconstrained Total Elbow Arthroplasty,” Journal of Bone and Joint Surgery. American Volume 82, no. 10 (2000): 1379–1386. 11057465 10.2106/00004623-200010000-00003

[35] I. Voloshin , D. W. Schippert , S. Kakar , E. K. Kaye , and B. F. Morrey , “Complications of Total Elbow Replacement: A Systematic Review,” Journal of Shoulder and Elbow Surgery 20, no. 1 (2011): 158–168. 21134667 10.1016/j.jse.2010.08.026

[36] M. D. Mckee , D. M. Pugh , R. R. Richards , et al., “Effect of Humeral Condylar Resection on Strength and Functional Outcome After Semiconstrained Total Elbow Arthroplasty,” Journal of Bone and Joint Surgery. American Volume 85, no. 5 (2003): 802–807. 12728028 10.2106/00004623-200305000-00005

[37] A. Celli , C. Paroni , P. Bonucci , and L. Celli , “Total Elbow Arthroplasty for Acute Distal Humeral Fractures With Humeral Condyle Resection or Retention: A Long‐Term Follow‐Up Study,” JSES International 5, no. 4 (2021): 797–803. 34223433 10.1016/j.jseint.2021.03.006 PMC8245998

